# Tachycardia with a shock – what is the underlying mechanism?

**DOI:** 10.1016/j.ipej.2026.04.002

**Published:** 2026-04-06

**Authors:** Deva Nirthanakumaran, Belinda Gray, Bianca Coelho, Hari Raju

**Affiliations:** a Department of Cardiology, Macquarie University Hospital, Australia; b Department of Cardiology, Royal Prince Alfred Hospital, Sydney, New South Wales, Australia; c Department of Cardiology, Concord Repatriation General Hospital, Sydney, New South Wales, Australia; d Faculty of Medicine and Health, University of Sydney, New South Wales, Australia

## 1 Case

A 43yo woman presented with two episodes of syncope and head strike following implantation of a primary prevention dual chamber implantable cardioverter defibrillator (Boston Scientific Resonate, implanted March 2024, Leads: 7842 atrial lead and 0672 ventricular lead). Device interrogation revealed two instances of ventricular arrhythmia each terminated successfully by a single 41 J shock (Fig. 1).

**Fig. 1:**
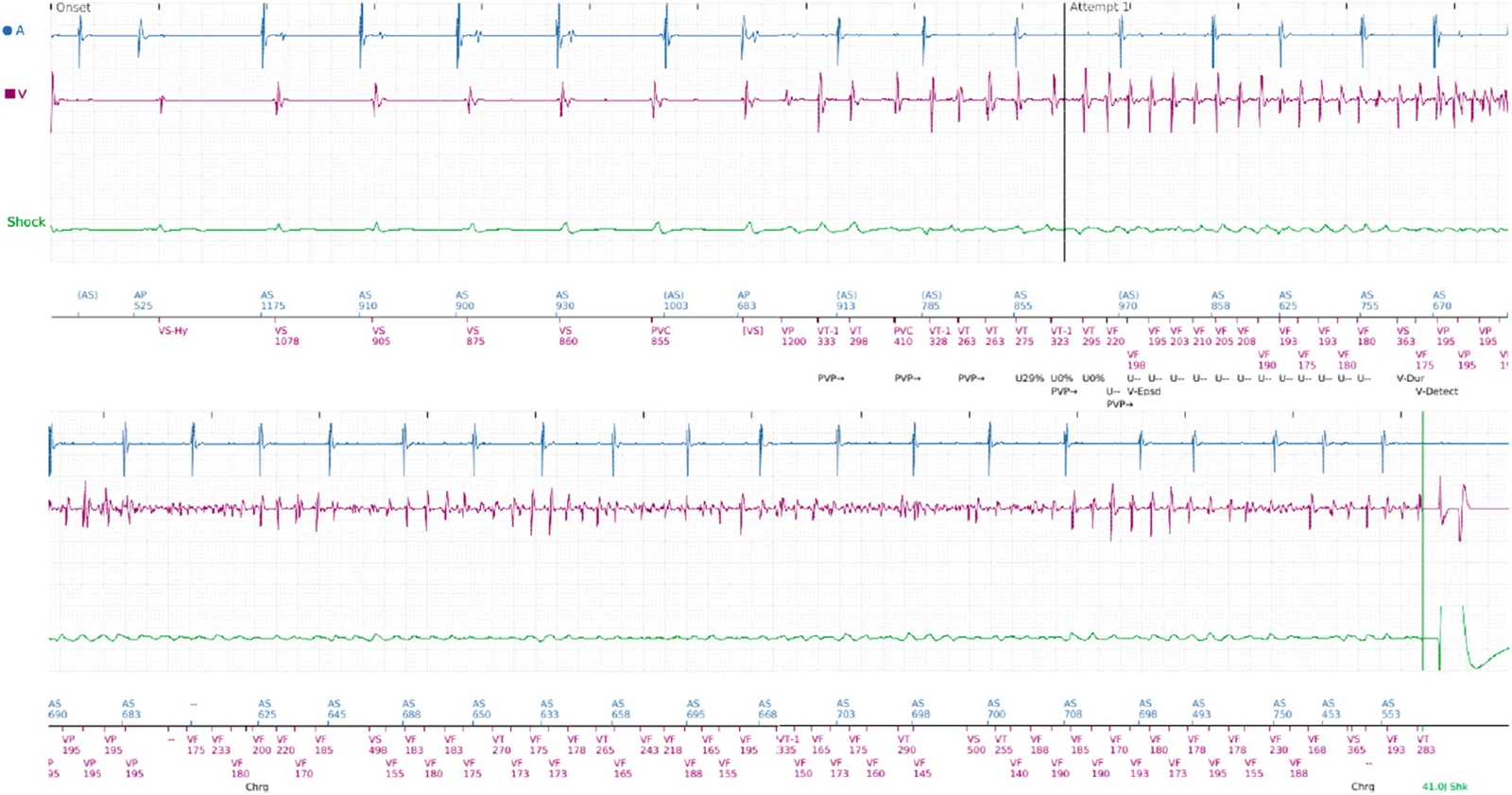
Stored electrogram of syncopal event resulting in delivery of single 41J shock.

What is the precipitating mechanism of her ventricular arrhythmia, and what programming changes should be made?

## 2 Discussion

On the EGM a premature ventricular contraction (PVC) is sensed within the blanking period immediately following a paced atrial beat; a subsequent paced ventricular beat at the lower rate interval (excluding the blanked PVC) at coupling interval of 355 ms initiates polymorphic ventricular tachycardia (VT). Thereafter, there is further degradation into an irregular, even faster (cycle length range 150 – 200 ms) ventricular rhythm consistent with ventricular fibrillation (VF) which is terminated with a single 41 J shock. The above sequence of events is confirmed on scatter plot as shown in Fig. 2. Notably, the rhythm up to 4 seconds prior to the initiating paced ventricular beat is likely idioventricular with isorhythmic dissociation evidence by VV intervals just shorter than preceding AA intervals (including a blanked A sense event) without Wenckebach behaviour.

**Fig. 2:**
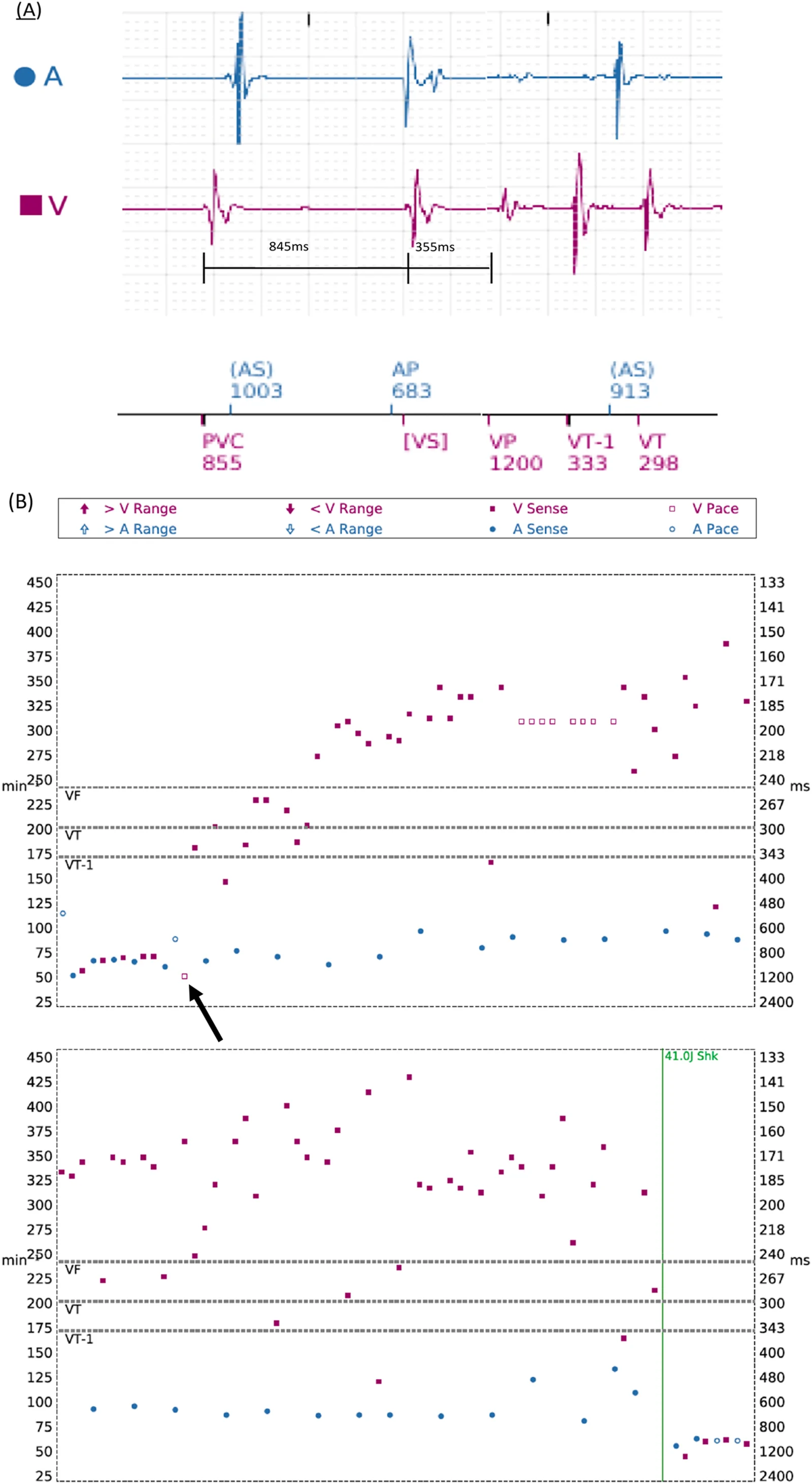
(A) Focused view of intracardiac electrogram which illustrates the erroneously long reported coupling interval of the paced beat (reported at 1200 ms), with correct callipers overlayed to measure intervals involving the immediately preceding blanked ventricular contraction [VS]. (B) Scatter plot demonstrating the paced ventricular beat (black arrow) which precipitates VT degenerating into the VF zone resulting in delivery of a single successful 41J shock. The blanked ventricular contraction [VS] prior to onset appears to be part of an idioventricular rhythm with isorhythmic dissociation (ventricular cycle length 855 to 905 ms), and is not annotated on the scatter plot.

The critical precipitating issue is the occurrence of a ventricular ectopic beat within the ventricular blanking period post atrial pacing. The paced beat occurs during the post PVC repolarisation phase with an intermediate coupling time of 355 ms, leading to VF due to the R on T phenomenon [1]. Given an unknown QT interval, another differential includes initiation by a late-coupled PVC leading to re-entry ventricular tachycardia.

A ventricular blanking period is initiated each time the atrium paces, to avoid erroneous sensing of the far-field atrial stimulus as a ventricular impulse. In the Boston Scientific Resonate dual chamber ICD, the minimal post atrial pacing ventricular blanking period is 45 ms (default 65 ms); we believe even this duration is insufficient in the above case to prevent the blanking of similar ectopic beats. The post-atrial pacing ventricular blanking period is described in the four major international device manufacturers (Table 1), and cannot be removed or programmed to a sufficiently low level to have prevented these events from occurring.

**Table 1:** Comparison between the four main international device manufacturers and default and minimal programmable (when applicable) post-atrial pacing ventricular blanking periods.

Device Manufacturer	Post-atrial ventricular blanking period		Further Notes
	Default	Minimum	
Boston Scientific	65 ms	45 ms	Programmable from 45 to 125 ms in pre-selected increments
Biotronik	50 ms	40 ms	Programmable from 40 to 100 ms in pre-selected increments
Medtronic	30 ms	30 ms	Non-programmable. When in ADI-DDD and there is an A paced – V sensed event, the post-atrial pacing ventricular blanking period shortens to 10 ms
Abbott	44 ms	44 ms	Non-programmable.

Given that this patient did not have a pacing indication, the device was reprogrammed from DDD 50/130 to VVI 30 to eliminate triggered ventricular pacing in the high-risk repolarisation phase following atrial pacing that coincides with intrinsic ventricular beats.

Alternate programming strategies were explored to reduce recurrence risk. These included shortening the paced AV delay and disabling AV Search+, which would work albeit at the cost of increased RV pacing burden. A RHYTHMIQ™-based strategy with a short AV interval would permit non-conducted p waves, but otherwise pace at a fixed AV interval. Given the absence of a pacing indication, reprogramming to VVI 30 bpm was considered the most definitive strategy to eliminate pacing-mediated arrhythmia.

Most documented cases thus far of pacing induced R on T phenomenon occur in the setting of under-sensing [1]. We present the unique situation of sustained polymorphic VT and VF requiring defibrillation due to a paced ventricular beat occurring in the vulnerable repolarisation phase of a premature ventricular beat that was not sensed as it fell in the post atrial ventricular blanking period.

## Editorial Commentary (Kartikeya Bhargava)

The authors report a case wherein a ventricular arrhythmic event was initiated by a Vp event that occurred after the device did not see an intrinsic ventricular event (a PVC) falling in the post atrial ventricular blanking (PAVB) period. While analyzing the EGMs in a device, it is essential to understand the marker channels used by the specific manufacturer. In the Boston Scientific devices, an event falling in the blanking period is marked in rectangular brackets whereas an event falling in the refractory period is marked in parentheses. For example, the atrial event before the Ap fell in the Post Ventricular Atrial Refractory Period (PVARP) and is denoted as (AS).Also, the device action differs in events falling in the blanking and refractory period. Notably, the blanked events do not initiate any action by the device although still shown in the EGM and annotations. Whereas the refractory events are counted by the device for various calculations and initiate their own refractory periods but do not reset the lower rate intervals or trigger response in contrast to non-refractory events. These differences are basic and uniform to all device manufacturers although depiction and annotations vary.The case shown also beautifully illustrates how deep dive into the electrogram analysis and can help in individualized changes in programming to do the best for the patient.

## Informed consent

Written informed consent was obtained for anonymized patient information to be published in this article. It has been uploaded as a separate document.

## Ethics approval

Our institution does not require ethics approval for reporting individual cases or case series.

## Animal welfare

This research did not involve the use of animals.

## Funding

This research received no specific grant from any funding agency in the public, commercial, or not-for-profit sectors.

## Declaration of competing interest

The authors declare that they have no known competing financial interests or personal relationships that could have appeared to influence the work reported in this paper.
